# The Happy Home: Ageing, Migration, and Housing in Relation to Older Migrants’ Subjective Wellbeing

**DOI:** 10.3390/ijerph20010106

**Published:** 2022-12-21

**Authors:** Micheline Phlix, Ann Petermans, An-Sofie Smetcoren, Jan Vanrie

**Affiliations:** 1 Faculty of Architecture and Arts, UHasselt—Universiteit Hasselt, Agoralaan, 3590 Diepenbeek, Belgium; 2 Faculty of Psychology and Educational Sciences, Vrije Universiteit Brussel (VUB), Pleinlaan 2, 1050 Brussels, Belgium

**Keywords:** subjective wellbeing, housing, older migrants, sense of home, ageing, migration, environmental mastery, intersectionality

## Abstract

(1) Background: With an increasingly diversifying ageing population, it is important to understand what ‘ageing well’ means to older adults with a migration background. Given older adults’ preference to age in place and declining mobility, housing is a significant place in later life. Therefore, this paper explores the influence of housing, migration, and age on older migrants’ subjective wellbeing, with attention to immaterial aspects such as a sense of home as well. (2) Methods: In-depth interviews with older migrants from various ethnicities (N = 22) were conducted. The data collection and analysis were led by an inductive and deductive approach through thematic analysis. (3) Results: The results point to the dynamic nature of age(ing) and the role of migration background in the subjective wellbeing of older migrants. The need for preserving one’s housing situation and environmental mastery in later life is highlighted. Furthermore, the relation and mutual influence of subjective wellbeing and sense of home is uncovered. (4) Conclusions: This study highlights the intersection of age, migration, and housing to the subjective wellbeing and sense of home of older migrants. In addition, influences on older migrants’ subjective wellbeing concern both a material (i.e., housing) and immaterial (i.e., sense of home, age, migration) base.

## 1. Introduction

The ageing population in Europe is growing not only in numbers but also in ethnic diversity [1]. One of the explanations for this growing group of older adults is the ageing of post-war guest workers and their spouses. During the 20th century, Belgium experienced multiple waves of incoming labour migration from several countries, such as Italy, Turkey, Poland, and Morocco [2]. Although the diversity of Belgium’s ageing population is an outcome of different migration flows, post-war labour migrants are one of the dominant groups, especially in former mining municipalities. Given the lack of research taking into account the intersection of migration and ageing [3] and the growth in numbers of this particular group [1], understanding what wellbeing in later life means to them is crucial. Especially considering the challenges, older migrants face due to their often vulnerable social-economic position [3]. Extensive research [4,5,6,7,8] reports the various influences on subjective wellbeing, such as social relations, income, religion, health, and culture (e.g., [5]). In research on wellbeing, various conceptualisations can be found (e.g., life satisfaction, quality of life, eudaimonic wellbeing) (see [4,9,10,11]). However, in general, subjective wellbeing refers to “*an overall evaluation of the quality of a person’s life from her or his own perspective*” ([5], p. 3), which is also how the concept is approached in this paper. Although subjective wellbeing is a general concern for ageing well regardless of ethnic background, previous research points out that the interpretation of wellbeing is culture-specific, and consequently, research on such interpretations in the context of ageing and migration is needed [3,5,12]. Therefore, in this paper, we will further explore the subjective wellbeing of older migrants in the context of housing.

Concerning the influence of age(ing) on older adults’ subjective wellbeing, research shows that older adults generally experience an increase in wellbeing [4,8], despite an increased possibility of age-related challenges (e.g., declining mobility) [6,8]. Ryff [7] showed that older adults cope with change in a more positive way than their younger counterparts. However, this rise in subjective wellbeing strongly declines in very old age (80 and older) [4], which is possibly explained by feelings related to approaching the final stage of life [13].

Ryff introduced six dimensions of wellbeing; autonomy, environmental mastery, personal growth, purpose in life, positive relations with others, and self-acceptance [7,14]. Especially environmental mastery is interesting in relation to housing. This refers to one’s mastery over and competence in managing the environment [7,14], affecting older adults’ wellbeing [15]. Research has shown environmental mastery increases with age and that minority status is a significant positive predictor for environmental mastery [11]. Other factors that influence subjective wellbeing (e.g., social relations, housing) also vary with age [8,16].

Migrant or minority status is also related to subjective wellbeing [17]. However, research seems divided on the nature of its influence. On the one hand, migrant status has been observed as a positive influence on wellbeing [11]. This is because ethnic minorities tend to have more positive ageing perceptions [10], which have an effect on wellbeing [18]. On the other hand, migrants are likely to face ethnic inequalities, resulting in disadvantages which affect migrants’ subjective wellbeing. In addition, the proximity of ethnic peers enhances migrants’ subjective wellbeing [9]. Research has shown the importance of keeping in touch with family in the country of origin on older migrants’ wellbeing [17,19]. A positive attitude of the mainstream population towards migrants in the country of settlement has a positive influence on migrants’ wellbeing [20]. In addition, limited knowledge of the dominant language of the country of settlement negatively affects migrants’ feelings of belonging and wellbeing [19]. Closely related to migration is the role of culture, which is another important association with subjective wellbeing [5,20,21]. For example, subjective evaluations of wellbeing are dependent on people’s culture [5].

Housing fulfils a basic need and is thus associated with wellbeing [22]. Housing type, conditions, size, preferences, interior, and homeownership have all been associated with subjective wellbeing [5,23,24,25,26,27]. Looking at housing in later life specifically, research shows that housing that is adjusted to the needs of older adults promotes autonomy and contributes to their subjective wellbeing [24], but that renting can negatively affect older adults’ sense of environmental mastery [28]. Besides the house as such, the neighbourhood is another setting that is important to how older people evaluate their quality of life [24,29], as neighbourhood amenities enable older adults’ mastery [24]. Moreover, older adults tend to spend more time in their housing and immediate surroundings as they age. A study on older adults from various ethnic backgrounds reports that, on average, older adults spend 72% of their week inside their houses [30]. Moreover, most of them prefer to age in place (i.e., the wish to remain at home as one ages). However, a majority tends to live in inadequate housing (e.g., poor accessibility), hindering their possibility to age well in place [31]. Living independently at home comes with challenges (e.g., housing maintenance) which can also negatively impact their wellbeing [32]. Here, it is especially interesting to explore subjective wellbeing as older adults’ residential reasoning concerning the choice to age in place is affected by subjective experiences rather than (solely) by objective aspects [33]. This can explain why most older adults in Belgium wish to age in place regardless of the often—objectively—unsuitable dwelling [31]. Looking at housing in relation to migration, a recent Belgian study points to the lack of knowledge of how migrants live in the existing housing stock and stresses the importance of architects to be able to take migrants’ needs into account in design (i.e., diversity-sensitive design) [34]. Another study refers to a similar concept; culturally-sensitive housing. The authors pointed to the many culturally imbued activities that take place in the house (e.g., cooking, family values, religious practices) and influence wellbeing [35].

Closely related to housing is ‘home’. Home concerns a relationship with place, imbued with culture, derived from feelings of security, familiarity, and control. Although home has some material foundation, it goes beyond solely housing. Yet, home should not be approached separately from housing [36]. Closely related to this, recent research shows the effect of a sense of home on older adults’ wellbeing [37]. A sense of home entails a gradually developed [38] feeling of belonging [39] which concerns both material (e.g., physical environment, personal belongings) and immaterial attributes (e.g., social relations, memories) [37,38,40]. Boccagni pointed to the dynamic nature of constructing a sense of home parallel to changing life circumstances, which he conceptualised as ‘homing’ [36]. For older migrants specifically, a sense of home could be complex as they continuously renegotiate a sense of home in both the country of origin and settlement [41,42].

In sum, research on older migrants [3,42,43,44] and their subjective wellbeing in relation to housing is needed but remains scarce. For example, in a recent scoping review of Pani-Harreman et al. on ageing in place [45], migration or ethnicity is not mentioned. Therefore, this paper explores the intersection of ageing, migration, and housing in relation to older migrants’ subjective wellbeing (RQ1). Additionally, given the close relation to a sense of home, this study explores in what way a sense of home relates to subjective wellbeing (RQ2). Finally, considering the focus on older migrants, this study explores the influence of age and migration on the subjective wellbeing of older migrants (RQ3).

## 2. Materials and Methods

### 2.1. Recruitment, Participants, and Data Collection

We conducted 19 in-depth interviews with 22 older migrants (3 couple interviews). The search criteria were: (1) aged 60 and older, (2) 1st, 1.5 (people who migrated at a very young age, as children) or 2nd migration migrant, (3) living at home in Limburg, Belgium. Limburg is a province in Belgium known for its rich labour migration history. Following the discovery of coal in the 20th century, several mines opened and recruited foreign labour forces from several countries such as Italy, Poland, Spain, Greece, Turkey, and Morocco. Participants were recruited in various ways; through the network of the researchers established by previous interviews with older migrants, through ‘snowballing’, and with the help of municipality and community workers. This resulted in a diverse sample of 22 participants, which included 8 men and 14 women of Italian (N = 10), Polish (N = 4), Moroccan (N = 2), Dutch (N= 2), Turkish (N = 1), Russian (N = 1), Spanish (N = 1), and Greek (N = 1) migration background (see Table 1). All interviews were conducted in Dutch. Most interviews were conducted in participants’ homes (N = 12). In some cases, an interview day was organised by the community worker, in which case the interviews took place in the local community centre (N = 7). All interviews took place in Genk and Maasmechelen, former mining municipalities in the province of Limburg (Belgium). All participants signed informed consent and were given the option to choose a pseudonym themselves. Some participants did not want to use a pseudonym and preferred to use their first name, which we adhered to in this paper.

### 2.2. Researcher–Participant Positionality

In terms of researcher–participant positionality, the first author would be considered an ‘outsider’ [46] (i.e., young, Belgian origin). However, being an insider is not a necessary condition to adequately represent participants’ experiences [47]. A recent study points out several disadvantages to involving researchers of the same ethnic origin in research with older adults with a migration background (e.g., taking cultural knowledge for granted). In preparing the recruitment, data collection and analysis, the authors paid specific attention to the possible effects of researcher–participant positionality (e.g., reading up on the topic and drawing on previous research experiences with older migrants). For example, occurrences during the interviews where participants explicitly positioned themselves in relation to the researcher or a group she belongs to (e.g., young, white) were coded (e.g., “*When you get older, you feel it in your joints. You don’t notice it yet at your age, but at my age, you start to feel it*”).

### 2.3. Interview Guide

Based on the literature, a semi-structured interview guide was set up. Because of the extensive research on subjective wellbeing and its measurements, Ryff’s framework [7,14] was used as a sensitising concept, a theoretical idea derived from the literature review [48,49] that can outline important enquiries [48]. Here, Ryff’s textual descriptions of dimensions of wellbeing formed the basis to further develop part of the interview guide. As the interpretation and conceptualisation of subjective wellbeing can differ cross-culturally [5], the interview guide first queried older migrants’ personal interpretation of subjective wellbeing and sense of home. To do this, the interview guide consisted of open-ended questions. Next, we derived topics from the textual descriptions of every dimension in Ryff’s model for our interview guide (see Appendix A). Based on these topics concerning subjective wellbeing, and our additional interest in a sense of home, we formulated questions. In the interviews, we did not use the term ‘subjective wellbeing’ but ‘happiness’, which is more intuitive and colloquial language [50,51].

### 2.4. Data Analysis

The interviews were audio-recorded and transcribed (in Dutch). Afterwards, thematic analysis [52] was used to code all interviews in MAXQDA software (in Dutch). Here, we combined an inductive and deductive approach. Regarding the deductive approach, analyses partially departed from Ryff’s framework on dimensions of wellbeing [7,14] as a sensitising concept [48,53]. The pre-existing descriptions of every dimension became an initial code. In addition, the data were approached inductively as well. This meant coding every data extract relevant or meaningful to the research questions. Next, the coded extracts and code descriptions were revisited and revised where necessary. After this first round of coding (both inductively and deductively), themes started to emerge and were further explored [52]. Here, the use of visual tools (e.g., mind map) helped the exploration of relations between codes and themes. This resulted in 9 (preliminary) themes, including one theme of codes that did not belong to any of the other themes [52]. Next, the preliminary themes were reviewed and further refined (i.e., defining and naming themes, writing an analysis for every theme, and positioning every theme in relation to the research questions) [52]. Reworking the initial 9 themes, resulted in 3 overarching themes concerning the research context and material and immaterial influences on subjective wellbeing.

## 3. Results

In this section, first, aspects specific to the study’s research context (i.e., age and migration) are discussed. Second, material influences are discussed, focusing on housing and environmental mastery within the built environment. Third, moving beyond the built environment, older migrants’ sense of home in relation to their subjective wellbeing is addressed.

### 3.1. Research Context: Age(ing) and Migration

As older migrants are the target group of this study, participants were asked (both directly and indirectly) about their views on age(ing) and migration in relation to subjective wellbeing. Two issues came to the fore; the dynamic nature of age(ing) and the influence of migration background. As this paper is interested in older migrants’ subjective experiences, participants were asked about the possible role of their migration background in their happiness. In doing so, we aim to better our understanding of to what extent migration background can explain variances in subjective wellbeing.

#### 3.1.1. The Dynamic Nature of Age(ing)

The interviews highlighted the dynamic nature and the strong influence of age(ing) on various aspects of subjective wellbeing. Participants discussed changes in their views on ageing, life goals, and housing needs as they grow older. For example, some participants still expressed specific, new goals, such as learning a new language, taking computer lessons, or getting a driver’s license, or formulated more generic goals, such as being happy and enjoying life. However, many participants no longer had specific goals in later life.

“*When you are young, you still have a future, a goal. But at my old age, what am I going to achieve?*”

—Antonia, 66, Spanish migration background

Importantly, for most of these participants, this lack of specific goals in later life did not seem to affect their subjective wellbeing, as they had already achieved their main life goals. These participants also had a positive view on their past (e.g., sense of accomplishment, accepting ups and downs in life) and present life (e.g., being happy with the current situation). The interviews showed that satisfaction with achieved life goals contributes to participants’ subjective wellbeing.

“*I have no more goals. Look, I am done with my job. I am happily married. I had two children, and three grandchildren. That is fulfilled for me. Finito. There’s nothing more to add.*”

—Giacomo, 75, Italian migration background

Participants also discussed how their housing wants and needs (might) change as they age. For example, all participants expressed their wish to age in place. However, faced with their ageing body, an unchanged environment can now become an obstacle. What once was experienced as adequate housing becomes challenging in later life. Below, individual differences between husband and wife become apparent in their conversation. Unlike Jan, Lotka experiences the stairs in their split-level house as a struggle.

LOTKA: “*If I want to have breakfast outside, and I do, I drag everything downstairs, seven times, and then I have breakfast outside. If only I didn’t have to climb the stairs. That’s something you realise with age*.”

JAN: “*Now, now, now… with her illness*.”

LOTKA: “*Yes, but Jan, there will come a time when you won’t be able to march up the stairs cheerfully either.*”

JAN: “*I don’t have to be cheerful. At 80, I can still get up the stairs. It’s only five stairs. If it were twenty stairs, it would be a sport. As long as I can still get up the stairs here. If it were possible, I would do that for her, ground flour, and she’d get a pool as well.*”

—Jan (75, Polish migration background); Lotka (72, Polish migration background)

The challenge of a change in the match between people and their environment does not only apply to housing but also to the neighbourhood. For example, Piero (66, Italian migration background) states that he would consider a move to the city centre when he gets older, as the neighbourhood he currently lives in has changed a lot and would possibly hinder his wish to age in place.

“*We used to have all kinds of things here too. We had a bank here, a chip shop, and several shops. That used to be a shop there, an alternative has come there, but it is not the same as that shop. All that has fallen away. Then when you are a bit older, like my parents, they had nowhere to go to withdraw money, for example, then they depended on me.*”

—Piero, 66, Italian migration background

In analysing the dynamic nature of ageing and, in turn, its influence on older migrants’ subjective wellbeing, life course experiences should be taken into account. The data show how life events affected or changed participants’ subjective wellbeing. For example, for some participants, retirement generated some financial concerns, especially in relation to a possibly increasing care need when getting older. For others, moving generated a longing for the previous dwelling while also kickstarting a new homemaking process. Such life course experiences not only affect participants’ subjective wellbeing at a particular point in time but can also have long-term effects. For example, being faced with health problems—for either participants themselves or a significant other—can change one’s view on what is needed in order to be happy in a particular place.

“*Because I have had two serious illnesses. The first was breast cancer, which was seven years ago now. Thank God I am well now. Two years after that, I had a heart attack. And you start thinking like, if you are healthy, then you can do anything. If you have experienced that, twice, death before your eyes, if you have seen that, you think, let me be healthy, and the rest will come naturally.*”

—Paola, 65, Italian migration background

#### 3.1.2. The Influence of Migration Background

The participants elaborated on generational differences between migrants. First-generation migrants reported great effects of migration background on their subjective wellbeing in the first years after migration. For example, language served as a moderating negative influence on migrants’ social relations, which, in turn, negatively affected their subjective wellbeing. Second-generation participants were more familiar with Belgian culture and learned Dutch at school. From their own experience, interviewees reflected on the differences they observed between their parents and themselves. However, despite being more embedded within Belgian culture and life, many second-generation migrants could still experience an effect of their migration background. They face dualities such as growing up with two or more cultures, speaking multiple languages and constructing a sense of home in multiple places. For example, like their parents, second-generation participants struggled with the Dutch language. However, as they were very young, they were able to pick it up faster than their parents. Yet, it is striking that second-generation participants mention and still to this day recall how they experienced the linguistic struggle as a child. Another example of how the influence of migration trickled down through second-generation participants’ parents’ concerns accumulated disadvantages, not only because of linguistic challenges but also because of financial and educational challenges. For example, some participants shared that their first-generation parents were not able to support them with schoolwork and that their parents were not familiar with the Belgian school system. Second-generation participants elaborated on their experiences of being lower educated. Some participants also report dropping out of school early.

“*What I regret is that our parents didn’t know about education, for example, which schools to send us to. Now, we say, ‘those schools are better’, or ‘this is how we can help our children’. We didn’t have that. So we did miss those chances.*”

—Fatma, 62, Turkish migration background

“*I only went to school until I was fourteen, while I really liked going to school, and I really liked studying. I always was first or second in class. I really liked doing it, but financially at home, we were hungry, so I thought, I’m going to work so my mother would have a little more money.*”

—Antonia, 66, Spanish migration background

### 3.2. Material Influences on Subjective Wellbeing: The Built Environment

The results highlighted two material influences on subjective wellbeing. The first is older migrants’ dwelling itself and their wish to remain there for as long as possible, pointing to a need for housing stability. Second, and related to the first, the importance of environmental mastery in the ways in which participants interact with their environment while (physically) ageing, is discussed.

#### 3.2.1. Housing: Staying Put

In discussing how housing contributes to subjective wellbeing, homeownership often came up. On the one hand, being a homeowner (a cultural ideal in Belgium, promoted by the government for over decades) appeared to be an important life goal of many participants and, therefore, if achieved, positively contributes to subjective wellbeing. On the other hand, the current housing was important to older migrants’ wish to age in place. Most participants want to remain in their house for as long as possible, partly because they are very satisfied with their current dwelling due to various reasons, including the memories made there, the good location, familiarity, and a good relationship with neighbours. Consequently, they do not want to move (anymore). Moreover, they are willing to facilitate their strong wish to age in place, for example, by making housing adaptations, hiring cleaning staff, or arranging home care.

To further explore the relationship between housing and subjective wellbeing in later life, participants were asked about their views on residential care facilities and how they think moving there would affect their happiness. Overall, participants had a negative view of residential care facilities. Some participants explicitly said they would not be happy living there. Several reasons were mentioned: (1) loss of independence and dignity, (2) staff shortages made participants fear they would merely be a number or not receive good care, (3) loss of social interactions, (4) too expensive, (5), spatial concerns (e.g., too small), and (6) not enough consideration for migration background (e.g., not paying attention to differences in language and religion).

“*I sometimes visit a residential care home. Then when I return home, I think ‘no’. I don’t like that people living there are addressed with: ‘granny, do you want to drink?’. Granny! That person is not their grandma. That person has a name! It’s all good but those are the kind of things that offend me.*”

—Krysia, 85, Polish migration background

“*Your parents, when they are old, you don’t send them to a residential care facility. With us in Islam, the parents get tremendous respect and certainly the mother […] When you grow up, 40 or 50, you can’t leave them behind. You have to take them with you […] No. I don’t have my freedom there, and when I am with the family, I am happy. For example, in the residential care home, you have to eat what they serve, then the food is not hot, and the meat is cooked with alcohol. Then I would have to ask my son to take me to the mosque. I hope he keeps me here, really.*”

—Marouan, 65, Moroccan migration background

Although no participants expressed a strongly positive view on residential care facilities, some did see benefits (e.g., good to have the option, being taken care of, comfortable). Important to note is that these benefits are rather practical such as receiving good care. However, receiving quality care does not necessarily positively affect all participants’ subjective wellbeing, as demonstrated by Massimo’s quote below.

“*We have that support in Belgium. If you cannot live at home anymore, you have to go there because you have more comfort … A residential care facility is comfortable to the end. We are going there anyway, all of us. That is a place where you meet your end. You know that. Okay, we can stay alive longer there, in comfort, but I am not happy anymore.*”

—Massimo, 64, Italian migration background

#### 3.2.2. Environmental Mastery

Besides housing itself, the ways in which participants deal with the materiality of their house is a major influence on their subjective wellbeing. Having a sense of environmental mastery (i.e., a sense of control and independence within their own dwelling) was considered important to most participants in order to be happy. They elaborate on how they (try to) control their environment and (try to) adapt it to their wants and needs. Participants who rented their dwelling referred to their limited environmental mastery. For example, Liliana (76, Polish migration background) shared that she had to wait months before she was assigned a ground-floor apartment by the social housing corporation. Moreover, the effect of renting could also extend to participants’ subjective wellbeing. For example, Paola (65, Italian migration background) says, “*I would have loved to have my own house with a fireplace. Then I would be truly happy […] But the house isn’t mine*.”

In addition, one of the interviewees shared a story of when she faced health problems and turned down the offer of household help. She did this because she did not like a stranger to ‘grab’ her personal belongings. Several examples from the interviews will be used to further discuss the importance of environmental mastery to older migrants’ subjective wellbeing. In many instances, this is discussed in relation to feeling less independent (e.g., declining mobility as one ages) and the continuous process of balancing this contradiction within their housing and environment. Lotka’s quote below shows how she is trying to navigate this delicate balance between her mind (high independence) and body (declining independence). She has recently been struggling with her health, and besides the physical challenges, she also faces difficulties in dealing with the feelings associated with these physical challenges.

“*I like being independent. I don’t like to ask or delegate. I’m starting to learn because of those stairs […] Now I ask him to get me some potatoes from downstairs, but before that, I was exhausted and still went to fetch them myself. My husband says I should just ask. When I was lying here with my [broken] foot, I felt terrible. I was thirsty, but I was too proud to ask. He has to ask me that because I am help-dependent.*”

—Lotka, 72, Polish migration background

The interviews also point to the importance of participants’ subjective experiences in navigating their (in)dependence. Krysia’s quote below, for example, reveals how she harmonises her subjective experiences of dependence and independence. When asked about the meaning of subjective wellbeing (or happiness), Krysia mentions the importance of being independent. In further conversation, she recognises that, objectively, her independence is declining. However, subjectively, she does not experience it in that way. In fact, receiving help even contributes to her subjective wellbeing. This points to a delicate, complex balance between (in)dependence and subjective wellbeing.

“*For me, happiness is not being a burden to anyone for now. I think that is a bliss. Look, I don’t like driving at night anymore. I can’t see properly anymore. That’s old age. When I go to the eye doctor, he says I have old eyes. Then I am happy when I don’t have to drive and when someone drives. Small things make you happy, those little things. My granddaughter comes and helps me with the computer, little things like that. Then I do depend on them a little bit, but I don’t experience it in that way yet.*”

—Krysia, 85, Polish migration background

In the context of environmental mastery, physical health seems to be a strong influencer on the balance between participants’ perceived (in)dependence and subjective wellbeing. For example, Liliana (76, Polish migration background) currently struggles with her health and finds it very important to be independent. Therefore, she wishes for her situation to stay as it is, as she fears that her independence might be compromised if her health was to deteriorate even more. Other participants also experienced this strong link between health and (in)dependence. It seems that certain factors such as the degree of intimacy, the people involved (e.g., in caring for the older adult), and the type of activity can account for differences in how (in)dependence is experienced. First, the degree of intimacy seems to affect how participants perceive a loss of independence. For example, for Krysia (see quote below), a physical intimacy issue, such as having to be washed by others, evokes negative feelings (e.g., experiencing a loss of dignity and feeling uncertain about the future). However, other losses of independence where no (physical) intimacy comes into play, such as Krysia being no longer able to drive at night, are experienced differently.

“*I saw my mother. My mother got washed by my eldest daughter. Often, I think when I am washing myself that I am not ready for that yet. How lucky am I not to be faced with that yet! Who will wash me?*”

—Krysia, 85, Polish migration background

This relation between health and (in)dependence also plays out within the physical environment. Here, the environment can further challenge this delicate balance. For example, Lotka’s interview shows how her environmental mastery was contested by the stairs in her house on various occasions.

“*I couldn’t walk to the toilet on the first floor. So, I dragged myself upstairs on my butt and got up and went to the toilet.*”

—Lotka, 72, Polish migration background

Second, the people taking over some responsibilities seem to affect participants’ experience of declining independence. Some activities are preferred to be executed by significant others (e.g., family members), while other activities are preferably taken care of by strangers (e.g., care professionals). Again, these preferences also differ individually as some prefer family members to take care of more intimate activities (e.g., washing), while others would prefer professionals to handle these.

“*I wouldn’t want my life to be determined by people practising a profession. Because exercising a profession, that is compulsory […] They don’t do it out of their free will or love towards you. Their work rhythm determines what you have to do […] Personally, I would see it differently. For example, if I have to pay 2200 euros a month in a residential care home, and then when I see the wages of my daughters, who work eight hours a day, I think I can perfectly pay someone to take care of me here with that same money.*”

—Theo, 70, Greek migration background

Besides Theo’s wish to be taken care of by someone with whom he can build a relation with, he also expresses a strong environmental mastery, taking matters into his own hands. Multiple participants expressed similar wishes. For example, taking a family member from the country of origin to take care of them or adapting the house. It shows their willingness to be in control of their own lives and environment in later life, also when it comes to who provides support.

Third, the nature of the activities in which help is required also plays a great role in how the loss of independence is experienced among participants. For example, many participants were open to hiring cleaning staff if they would not be able to take care of the maintenance themselves. Although they partially give up some of their independence in light of their strong wish to age in place, it is not experienced as a loss per se. In fact, many participants view this as an acceptable arrangement that allows them to remain in their homes for as long as possible. So, although they partially give up some independence, they still experience a sense of control over their living situation, again pointing to the subjective experience of environmental mastery.

However, the interviews also revealed many instances in which participants did experience a restriction in their independence. The ageing body was one of the main causes mentioned by participants. Lotka’s quote below shows how she struggles with her ageing body in contrast to her (unchanged) environment, negatively impacting her subjective wellbeing.

“*I can’t do anything on my own anymore, and that’s my biggest enemy. I have to walk with my rollator. I used to go without […] I am already exhausted after only passing two houses, and my friend lives in the third. I used to visit her three times a week to have a coffee chat, but now, I only go once a week because it stops me. I don’t like going down the street with the rollator, and it is too far on foot. Then I’m exhausted. Then I can put myself down in the middle of the street and cry because I’m in pain.*”

—Lotka, 72, Polish migration background

Besides the ageing body, financials also affected some participants’ independence. Although no participants expressed being in financial difficulties, almost all of them were aware of the possibilities and limitations of their pensions. Financial restrictions especially impacted their view on housing options. Many participants are concerned that moving to a service flat or care facility will be too expensive. This was particularly expressed by respondents with a partner. This is also one of the reasons why most participants have a negative view of residential care facilities. Some participants also pointed out their housing would need adaptations in order to support their wish to age in place. However, this is financially challenging to owners, and renters are constrained in their possibilities to do so. Therefore, participants’ perceptions of their possibilities in the housing market in later life seem limited.

“*The bathroom should be different, with a walk-in shower.*”

—Krysia, 85, Polish migration background

“*The stairs to the basement are the house’s handicap […] There is no other way. I won’t go to the children because they have their own lives [...] If I have to move, I will only go to a residential care facility [...] I wouldn’t know where else to go.*”

—Elena, 71, Italian migration background

### 3.3. Beyond the Built Environment: A Sense of Home

In analysing the data to explore the role of a sense of home in relation to subjective wellbeing, the interrelatedness of both concepts became apparent. First, participants often used the concepts ‘house’ and ‘(sense of) home’ interchangeably during the interviews. For example, considering the effect of age(ing) on migrants’ subjective wellbeing, the interviews revealed that not only did participants want to preserve their current housing situation, but they also wished to preserve their sense of home. In asking participants what a sense of home entails, they often referred to their housing. It points to the important material foundation of housing to a sense of home. However, in addition, participants also mentioned immaterial aspects (e.g., social relations, language, safety, comfort). Some participants found it difficult or even impossible to imagine a change in their sense of home. A couple of respondents explicitly mentioned that they would struggle with changes. This struggle seems to increase with age. Possibly, this is because most interviewees lived many years in their current housing and strongly expressed their wish to age in place.

“*I like living here. I have placed and organised everything here. I know ‘how’ and ‘what’. I go to the shops and I know these people, that’s a bond. I have been here for so many years, that’s very special. You know every stone in the street, and you don’t like change. That’s maybe the age. I see young people now […] that’s a different mindset. As you grow older, you don’t want to come out of your shell. You say, ‘I know that’ and you don’t want to lose that.*”

—Fatma, 62, Turkish migration background

Looking at the relationship between subjective wellbeing and a sense of home, many instances of them intertwining stand out, but data still point to two different and separate concepts. In addition to this, the data also reinforce the subjective nature of wellbeing and sense of home, sometimes creating very different and individual experiences and interpretations. Consequently, the data do not point to a straightforward relation between a sense of home and subjective wellbeing. Some participants consider a sense of home to lead to or be a condition for happiness, while others approached this relation the other way around, pointing to the importance of being happy so that one can feel at home. In addition to this, some participants explicitly mentioned their views on the relationship between both concepts or used them during the interview interchangeably. For example, when asked about feeling at home, participants would shift to the concept of ‘happiness’, while seemingly still referencing the same train of thought. The results also show that one’s sense of home (attached to the house) can become important in coping with adversity in life, as reflected in Nico’s quote below. It shows that even though his happiness and sense of home are not aligned, it does not necessarily impact both. Moreover, in Nico’s case, his stable sense of home even serves as a safe haven during difficult moments.

“*Last year, my wife received bad news from the hospital. That was a very unhappy period. But still, I am happy that we could open the door and enter our home. That’s moving on. I’m home. I have a home to return to, where we can discuss the problems. That is a sense of home.*”

—Nico, 69, Italian migration background

Second, the interviews also pointed out the dynamic nature of a sense of home. Not only life events but also ageing in itself can constantly renegotiate one’s sense of home and subjective wellbeing. For example, Krysia (85, Polish migration background) says she used to be open to moving to a care facility, but because of her own experiences while visiting such facilities, she has now changed her mind. Elena (71, Italian migration background) says she would consider a move to a care facility if necessary, but she is aware that her view on this might change. This changing view as one ages can also affect one’s sense of home. For example, Giacomo (75, Italian migration background) states that the feeling of being a stranger in his country of origin has increased with age. This also points to the importance of taking life course experiences into account: of course, for first-generation migrants, migration in itself is a major life event, and the interviews reveal how this affects participants throughout their life course, pointing to a dynamism of subjective wellbeing throughout life. For example, Renata (69, Italian migration background) shares that she was unhappy and did not experience a sense of home right after migrating to Belgium. However, over the years and because of her children, she started developing a dual sense of home, both in Belgium and Italy. Parallel to this, she experienced an incline in her happiness. In addition, Massimo’s (64, Italian migration background) interview shows the changing influence of migration throughout his life course. At first, he felt like a stranger in Belgium, describing his migration as traumatic. However, after meeting his wife, learning Dutch, and securing a job, he started to feel at home in Belgium. Parallel to this growing sense of home in Belgium, his sense of home in Italy started declining over the years. Here, the intersection of age, migration, and a sense of home becomes apparent.

## 4. Discussion

The brief literature review indicated that influences on wellbeing can vary across age [8,16] and cultures [5,6,54] and the importance of housing and the immediate environment to subjective wellbeing in general [5,22,23,24,25,26,27], but especially in later life, was also highlighted [24,29], but adjoining this, research on older migrants seemed scarce [3,42,43,44]. Therefore, this paper aimed to explore the influence of housing on the subjective wellbeing of older migrants. In addition, specific attention was paid to the role of age, migration, and a sense of home on the subjective wellbeing of older migrants.

Research interested in particular features (e.g., age, migration) risks over-emphasising the role of these features. For example, migration research only focusing on ethnicity. Researchers should be mindful of other characteristics [55,56]. Therefore, to discuss various influences on older migrants’ subjective wellbeing, we adopted an intersectional lens. Intersectionality refers to the intersection of various features (e.g., gender, age, ethnicity, health), which results in a unique position for every individual and departs from the idea that different features come to the foreground in different situations, and some then also become irrelevant. This approach will allow us to reflect on the role of (the interrelatedness of) various features (e.g., migration background, age, housing) on the subjective wellbeing of older migrants. Below, we further discuss the results from different angles; (1) age, (2) migration background, (3) housing, and (4) the intersection of age, migration, and housing.

Our results pointed to the dynamic nature of ageing in relation to subjective wellbeing. This is in line with previous research, stating that influences on subjective wellbeing can vary with age [8,16]. Overall, participants in this study reported high levels of subjective wellbeing, despite facing age-related obstacles (e.g., declining health). It is important to note that all participants were able to fulfil their current wish to age in place, and the majority indicated that they were in relatively good health. As research shows, this trend of high levels of subjective wellbeing, despite facing age-related adversities, experiences a sharp decline in subjective wellbeing in the final stages of life [4,57]. In this study, participants reported similarly high levels of a sense of home in later life, despite facing—what objectively might be considered—an increase in negative home experiences (e.g., inadequate housing, facing dilemmas about ageing in the country of settlement or country of origin). However, when participants were asked about a change in their sense of home, they strongly indicated their preference to preserve their current sense of home and housing situation. Concerning this intersection of housing and age, participants reflected on moving to a residential care facility when asked about a possible future change in their sense of home. Most participants expected this to negatively affect both their sense of home and subjective wellbeing. Some even considered it to be impossible to experience a sense of home and be happy in a residential care facility. Therefore, we believe that this paradox of ageing and subjective wellbeing also extends to a sense of home; participants express an increasingly strong sense of home over time, peaking in later life but threatened by a change (e.g., moving to a residential care facility) that could drastically bring down their sense of home. Given the interrelatedness of subjective wellbeing and a sense of home, we argue that such a decline in older adults’ sense of home could negatively affect their subjective wellbeing. The dynamic nature of ageing also reflected to participants’ purpose in life, which is one of Ryff’s [7,14] dimensions of eudaimonic wellbeing. However, despite many participants explicitly expressing they do not have specific goals in life (anymore), it did not fit Ryff’s description of ‘low purpose in life’ as these participants’ interviews did not reflect a lack of ‘meaning in life’ or ‘direction’ ([14], p. 12). In this study, it seems that no longer having specific goals in life does not necessarily harm older migrants’ subjective wellbeing, precisely because their obtained goals in life result in a feeling of accomplishment. In addition, the results also pointed to the dynamism of environmental mastery in later life. Here, it is important to consider the subjective experiences of older adults as this can be different from the objective situation (e.g., not experiencing a loss of independence as such). Consequently, the impact on subjective wellbeing can also differ. Possibly, such subjective experiences could serve as a safety net for a decline in subjective wellbeing.

Besides ageing, the influence of migration background was also analysed in this study. The results show that it is valuable to consider the intersection of migration and age in understanding older migrants’ subjective wellbeing, as it can account for some variances. For example, the accumulation of disadvantages due to migration background was mentioned by some participants during the interviews. Research shows that this can have a negative influence on both subjective wellbeing [9] and the creation of home [41]. Taking the role of migration background into account has also pointed out that generational differences can account for variances in the effect of migration background on subjective wellbeing. This is in line with previous research [58]. However, this study also pointed to similarities across generations, such as experiencing a linguistic barrier. Previous research also points out that the role of culture—which is considered to influence people’s subjective wellbeing [5,20,21]—also affects second-generation migrants’ subjective wellbeing [20]. Of course, considering our diverse sample, the role of culture varied across participants. For example, regarding religiosity, mainly practising Muslim participants discussed the lack of attention to different cultures in the Belgian residential care facilities they knew. Looking at the intersection of migration and housing specifically, research also highlights the importance of a religious fit with the environment [5]. For older Muslim participants, it seems that there is a lacking fit with Belgian care facilities (as opposed to a good fit at home). The results point to the negative effect of a discrepancy between one’s own cultural norms, goals and practices and the imposed culture of the country of settlement on older migrants’ subjective wellbeing. Often, the influence of migration background seems to be interrelated with other variables such as ageing, religion, etc. Because most of our participants have lived in Belgium for many years or were born here, the role of migration background on their subjective wellbeing seems to have shifted more to the background. Rather, in later life, the role of age(ing) seems to be more dominant than migration background. In reviewing the data, many themes and quotes are unrelated to migration or ethnicity. Therefore, we stress the importance of adopting an intersectional analytical approach in research on older migrants. This way, other relevant influences are not overlooked, and the role of migration background is not overemphasized.

This study specifically focused on housing, age, and migration in relation to subjective wellbeing. Here, the aforementioned dynamic nature of ageing was also highlighted in contrast to the environment (i.e., housing and the neighbourhood), and whether this did or did not change over time. A changing living environment can have different impacts on older migrants’ subjective wellbeing. Ageing in place can negatively impact older adults’ subjective wellbeing [32]. For example, some participants regretted losing amenities in the neighbourhood, such as shops and feared it could form a future obstacle in their wish to age in place, possibly obstructing their subjective wellbeing. In contrast, remaining in the familiar neighbourhood—despite the lack of amenities—was important to maintain participants’ sense of home. However, a change in environment can also positively impact subjective wellbeing. For example, Krysia (85, Polish migration background) shared that she is happy with her choice to move to an apartment. However, as opposed to high levels of subjective wellbeing, her sense of home is affected differently as she does not experience a sense of home to the same extent as in her previous house. Next, the house is also a place where cultural activities (e.g., practicing religion, cooking, organising family gatherings) take place, which in turn contribute to wellbeing [35]. Participants’ negative stance towards residential care facilities was, for some, also partially due to cultural discrepancies. In contrast, it seems that ageing in place also facilitates participants’ cultural practices.

Various housing options should be available to older adults. Hence, given the diversifying ageing population, (interior) architectural design should take this increasing diversity into account. Not only in terms of migration background and culture but also taking other sources of diversity into account (e.g., finances, language, gender). This is also in line with research on (design for) subjective wellbeing that points to the importance of a tailored approach fitting with the individual’s interests [59,60,61]. Furthermore, housing also closely relates to environmental mastery (one of Ryff’s dimensions of wellbeing). In managing the delicate balance between highly valuing and experiencing a decline in environmental mastery, the results demonstrated that participants exhibited three coping strategies; (1) changing their behaviour (e.g., using a rollator to be able to still go out for coffee with friends), (2) changing their attitude (e.g., experiencing the need of help as pleasant instead of unpleasant), and (3) changing the environment (e.g., moving towards a better-suited house for ageing in place). Considering these changes in later life, the results highlight the importance of flexible, age-friendly and adequate housing to both older migrants’ subjective wellbeing and their sense of home, as it facilitates ageing in place. The latter is preferred by all participants, partly due to their strong sense of home attached to their current dwelling. However, a challenged sense of environmental mastery threatens participants’ subjective wellbeing. Such adjusted housing also promotes subjective wellbeing and autonomy—also one of Ryff’s dimensions of wellbeing [7,14]—of older adults [24]. Closely related to housing, this study also explored older migrants’ sense of home and how this relates to their subjective wellbeing. Although both concepts are influenced differently, the results point to a close relationship between them, possibly influencing each other. Most participants consider the presence of a sense of home to be conditional or contributing to their subjective wellbeing, or happiness. The results show various ways in which a sense of home and subjective wellbeing are related to each other—which varies individually. Additionally, the study highlights the protective role of a sense of home in challenging times that negatively impact one’s subjective wellbeing.

Finally, the results point to the importance of other intersecting features, especially health and homeownership. Both can again be linked to control. A declining health can challenge one’s control over everyday life affairs in an unchanged environment (e.g., struggling with stairs). Related to this, renting a property can obstruct one’s sense of control over one’s own home (e.g., not being able to fully adapt the home to one’s changing needs). Control seems a key element in this as it is both a dimension of home [36] as well as wellbeing (i.e., environmental mastery) [7,14]. Hence, we argue that, besides age, migration, and housing, also health and homeownership affect older migrants’ subjective wellbeing and their sense of home.

## 5. Conclusions

Adopting an intersectional lens has shown that the intersection of age(ing) and housing is a prominent feature in relation to older migrants’ subjective wellbeing. Such an approach has enabled us to identify various and intersected influences on subjective wellbeing. For example, the results point to the importance of housing, especially at the intersection with age. It shows that an unchanged environment parallel to the changing needs in later life can become challenging and affect one’s subjective wellbeing. Although not absent, migration background as an influencing factor then seems to shift to the background in most instances—whereas this was the other way around for many first-generation participants right after migrating to Belgium. However, the results do point to some instances where housing, age, and migration background all intersect.

In addition to identifying the important role of housing, this study points to immaterial aspects, such as environmental mastery and a sense of home, that also affect older migrants’ subjective wellbeing. Therefore, future research could further explore how ‘home’ is experienced or sensed by older migrants, especially in relation to their subjective wellbeing. Besides exploring (the intersection of) various influences on older migrants’ subjective wellbeing, the results of this study highlight the importance of subjective experiences. In other words, although the identified influences can affect older migrants’ subjective wellbeing, this was not the case for all participants, highlighting the individual and subjective nature of happiness.

A limitation of this paper concerns the use of community workers to recruit some participants as this could result in a selection bias [62]. In this study, participants recruited by the community workers were actively involved as volunteers in the community centre. Therefore, to respond to this, a variety of other recruitment strategies (i.e., contacting previous participants, using the researcher’s personal network, snowball sampling) was used. Another limitation concerns the very specific labour migration context (i.e., mining) in which most participants (or their parents) migrated to Belgium, as this could impact the results. Our results point to the possibility of accumulated disadvantages impacting participants’ subjective wellbeing (e.g., missed educational opportunities). However, as most participants have lived in Belgium for many years, the impact of migration background on their subjective wellbeing seems to diminish over time. Parallel, the impact of ageing seems to come to the foreground over time, possibly challenging participants’ subjective wellbeing (e.g., being faced with their ageing body). However, migrants are exposed to many risks in the country of settlement that negatively impact their subjective wellbeing. Examples of such risks are housing insecurity [26,63] and discrimination. Such accumulated risks can negatively impact migrants’ subjective wellbeing [26] and their creation of home [41]. In this study, most participants had lived in Belgium for many years and the role of migration background and associated disadvantages has diminished over time. Hence, they self-reported relatively high levels of subjective wellbeing. However, we argue, if risks related to ageing intersect with accumulated risks due to migration background (e.g., in other migration contexts such as follow-the-children migration or international retirement migration), it could result in additional vulnerabilities for older migrants, possibly negatively impacting their subjective wellbeing and sense of home. Hence, it would be interesting for future research to explore older migrants’ subjective wellbeing in relation to housing and a sense of home in various migration contexts.

As this study only included participants living at home in relatively good health, it would be interesting for future research to further explore the role of housing in subjective wellbeing among various settings (e.g., co-housing, residential care facilities) and among older migrants with various care needs.

## Figures and Tables

**Table 1:** Overview of the participants in this study.

Participant (Age)	Migration Background and Generation	Housing Type	Renter or Owner
Nico (69)	Italian	House	Owner
	1.5 generation		
Renata (69)	Italian	Apartment	Renter (social housing)
	1st generation		
Marouan (65)	Moroccan	House	Owner
	1st generation		
Aicha (around 60 ^1^)	Moroccan	Apartment	Renter (social housing)
	1st generation		
Vera (62)	Russian	Apartment	Renter (social housing)
	1st generation		
Fatma (62)	Turkish	House	Owner
	1st generation		
Elena (71)	Italian	House	Owner
	1.5 generation		
Rietje (75)	Dutch	House (ground floor)	Renter (social housing)
	1st generation		
Giacomo (75)	Italian	House	Owner
	1.5 generation		
Krysia (85)	Polish	Apartment	Renter
	2nd generation		
Jan (75)	Polish	House	Owner
	1.5 generation		
Lotka (72)	Polish	House	Owner
	2nd generation		
Massimo (64)	Italian	House	Owner
	1st generation		
Isabella (59 ^2^)	Italian	House	Owner
	2nd generation		
Halina (71)	Polish	House	Owner
	1st generation		
Liliana (76)	Polish	Apartment	Renter (social housing)
	2nd generation		
Paola (65)	Italian	House	Renter (social housing)
	2nd generation		
Inge (63)	Dutch-Indonesian	House	Owner
	1st–2nd generation		
Mauro (65)	Italian	House	Owner
	1.5 generation		
Piero (66)	Italian	House	Owner
	1.5 generation		
Antonia (66)	Spanish	House	Owner
	1.5 generation		
Theo (70)	Greek	House	Owner
	1.5 generation		

^1^ Aicha’s exact age is unknown due to unclear birth registrations in Morocco. ^2^ Despite not formally meeting the age-related search criterium (i.e., aged 60 or older), Isabella provided valuable insights and was therefore included in the study.

## Data Availability

The data presented in this study are available on request from the corresponding author. The data are not publicly available to protect the privacy of our participants.

## Appendix A. Interview Guide

- Can you tell me a bit about yourself?
- What do you think of when you hear the word ‘happiness’?
- What do you need to be happy?
- What does ‘being happy’ mean to you?
  o Alternative/additional question: How would you define happiness for yourself?
- What do you consider your ‘home’?
- Can you tell me what a typical day in your life looks like?
- Do you experience a sense of control over your own home?
  o If yes/no, can you give a specific example of this?
  o Side question/alternative question: What do you have a lot/little control over in your own home?
  o How do you feel about this feeling of/lack of control?
- How would you react if someone made a (negative) comment about your home (e.g., garden, household)?
  o How much do you value the opinions of others?
- Can you tell me a little about the most important people in your life?
  o How would you describe your relationship with …?
- What place do(es) … **person mentioned before** have in your idea of home?
  o Prompt: ask about close (e.g., partner, children) and wider network (e.g., neighbours)
  o How would you feel if … **person mentioned before** did not live here/close by?
- Can you tell me in what way you would make an important decision about your home?
  o Can you give an example of such a decision?
- How do you feel about moving?
  o What would you think or do if you would consider moving?
- Have you ever experienced a major change in terms of your home?
  o If yes, can you tell me more about this?
  o If not, what does a major change in terms of your home entail to you?
  o How do you feel about changes to your home in the future?
  o Can you imagine a home in a different environment in the future?
- How do you feel about the life you have led so far?
  o How do you feel about the things you have already achieved in your life?
  o Can you give me a specific example of this?
  o What role did your home play in this?
- What are your (most important) life goals?
  o Do you feel you have achieved them?
  o Are there any other goals you would like to achieve in your life?
  o Does your home play a role in the extent to which you could/can fulfill these life goals?
- What do you need to be completely yourself?
  o Can you give me a specific example of this?
  o What role does your home play in this?
- In what way does your home play a role in your happiness?
- When you think about your home, what do you get pleasure, joy from?
- If you were allowed to design your ideal home now, what should definitely be present?
  o Thinking about your current home now, is there any difference from this ideal home? And if so, which difference(s)?
- Do you think migration background plays a role in the issues we have discussed today (i.e., happiness and home)?
- Is there anything else you would like to add?
